# How to eliminate taeniasis/cysticercosis: porcine vaccination and human chemotherapy (Part 2)

**DOI:** 10.1186/s12976-019-0100-x

**Published:** 2019-02-26

**Authors:** Norma Y. Sánchez-Torres, Juan R. Bobadilla, Juan P. Laclette, Marco V. José

**Affiliations:** 1 Theoretical Biology Group, Biomedical Research Institute, Universidad Nacional Autónoma de México, Ciudad Universitaria, 04510 CDMX, Mexico; 2 Department of Immunology, Biomedical Research Institute, Universidad Nacional Autónoma de México, Ciudad Universitaria, 04510 CDMX, Mexico

**Keywords:** Taenia-cysticercosis, Susceptible-infected mathematical model, Vaccination strategies, Chemotherapeutic interventions, Elimination, Eradication, Computer simulation experiments, Public health

## Abstract

**Background:**

The application of effective vaccines against pig cysticercosis and mass chemotherapy against pig cysticercosis and human taeniasis have shown the feasibility of interrupting the parasite’s life cycle in endemic areas.

**Methods:**

A mathematical model that divides the population into susceptible, infected, and vaccinated individuals is formulated. The model is based upon the life cycle of the parasite. Computer numerical simulation experiments to evaluate the impact of pig vaccination under different vaccination schedules, and combined intervention strategies including pig vaccination and anthelmintic treatment against human taeniasis are carried out.

**Results:**

Vaccination against either pig cysticercosis or against human taeniasis will influence the transmission dynamics not only among vaccinees but also the dynamics of the other hosts as well. When the protective efficacy and/or the coverage rate is less than 100%, different mass interventions like vaccinating the pig population twice in combination with chemotherapeutic treatment against human taeniasis, the elimination of the infection in both pigs and humans can also be achieved.

**Conclusions:**

Our mathematical model has the potential for planning, and designing effective intervention strategies including both mass vaccination and/or chemotherapeutic treatment to eliminate pig cysticercosis, human taeniasis and human neurocysticercosis. The model can be adapted to any given community with mild, moderate endemicity, or even in hyperendemic regions.

**Electronic supplementary material:**

The online version of this article (10.1186/s12976-019-0100-x) contains supplementary material, which is available to authorized users.

## Background

Tapeworms were among the first known parasites of humans, recorded by Hippocrates and Aristotle in ∼ 300 BC [1], but a safe and efficient cure to larval tapeworm infection in humans has yet to be found. Cysticercosis, is a severe parasitic disease in humans and account for 1 of the 17 neglected tropical diseases prioritized by the World Health Organization [2]. Larval tapeworms can persist asymptomatically in a human host for decades [3], eventually causing a spectrum of debilitating pathologies [4]. When diagnosed, the disease is often at an advanced stage at which surgery is no longer an option [5]. Tapeworm infections are highly prevalent worldwide [6], and their human disease burden has been estimated at 1 million disability-adjusted life years, comparable with African trypanosomiasis, river blindness and dengue fever. Tapeworms (Platyhelminthes, Cestoda) are passively transmitted between hosts and parasitize virtually every vertebrate species [7]. Their morphological adaptations to parasitism include the absence of a gut, a head and light-sensing organs, and they possess a unique surface (tegument) that can withstand host-stomach acid and bile but is still penetrable enough to absorb nutrients [7]. Tapeworms are the only one of three major groups of worms that parasitize humans, the others being flukes (Trematoda) and round worms (Nematoda), for which no genome sequence has been available so far. The genome of *T. solium* [8] offers an expanded armamentarium of tools to interrogate the biology of the parasite, identify molecules involved in host–parasite interactions, and discover more effective and safer vaccines and cysticidal drugs. The adult tapeworm (taeniasis) occurs only in humans and carries mild clinical manifestations or none.

The zoonotic tapeworm *Taenia solium* is formally recognised by the WHO as a Neglected Tropical Disease [9], and it is ranked first on the global scale of foodborne parasites by the Food and Agricultural Organization of the United Nations (FAO) [10].

The widespread disease is endemic in many parts of Latin America, Asia and Africa, with an increasing concern of case introduction in previously non-endemic countries through migration of human tapeworm carriers [11, 12]. Cysts in the human central nervous system can result in neurocysticercosis (NC), manifesting clinically as epilepsy, chronic headaches, vertigo, visual disturbances and nausea amongst other symptoms [11]. NC is the most common cause of adult-acquired epilepsy worldwide and one the most frequent parasitic infections associated with chronic morbidity in the United States [13]. NC is common in many countries of Central and South America, Haiti, India, most of Africa, Southeast Asia, and parts of China. Estimates of the burden of cysticercosis and NC, based upon detailed studies of endemic communities, have been reported [14], and the burden of disease is substantial [15]. In endemic regions, 29% of seizure cases are associated with NC [16]. It is the only common infectious disease or process where normal individuals are exposed *en masse* to a seizure-inducing agent [13].

Transmission of *T. solium* was eliminated in a highly endemic region in Peru [17] in a large-scale field trial, demonstrating the feasibility of interrupting the parasite’s life cycle. A final elimination strategy of mass chemotherapy with niclosamide in humans and with oxfendazole in pigs, in combination with pig vaccination (final mass treatment with vaccine), was implemented in all 107 rural villages in Tumbes Region over a period of 1 year [17]. Another smaller intervention trial, carried out in Cameroon [18], was successful in eliminating *T. solium* transmission by the pigs that were enrolled in the trial. Assana and colleagues [18] used both the TSOL18 vaccine and chemotherapy (oxfendazole) in piglets distributed to farmers in a *T. solium* endemic area. While the intervention in Cameroon was relatively simple and very successful, it involved a cohort of animals. For any intervention in pigs to be effective in eliminating *T. solium* transmission, it would need to be applied to the entire pig population and be both practical and sustainable. Typically, pigs breed throughout the year and new, *T. solium*-susceptible animals are born into the population continuously. This represents a major challenge for implementation of any intervention that seeks to prevent *T. solium* transmission by pigs.

The TSOL18 vaccine has proven to be the most effective vaccine against *T. solium*, with independent experimental vaccine trials carried out in Mexico, Peru, Cameroon and Honduras inducing 99.3–100% protection against an experimental challenge infection with *T. solium* eggs in pigs [19–21]. The results were the absence of cysticercosis at autopsy of pigs and a decrease in prevalence from 19.6 to 0.0001%.

The use of TSOL18 plus oxfendazole prevented any detectable infection with *T. solium* in pigs raised under natural conditions [18]. The effectiveness of vaccination (> 99%) reported in this trial is as high as the vaccine effectiveness found in studies that used controlled conditions [19, 20].

A combination of porcine and human chemotherapy was performed experimentally in northern Lao [22], applying a dose of abendazole for three days to the human and at the same time TSOL 18 vaccine to the pig was given along with oxfendazole. A month later, the combination of vaccine and chemotherapy in the pig was repeated. The combination of vaccine and chemotherapy in pigs and at the same time the use of chemotherapy in humans, reduced in a short period the taeniasis in the human [23].

The TSOL18 can provide almost completet protection against porcine cysticercosis [20–22] and is currently on-going registration processes for use in pigs in several countries [24]. The vaccine is by default assumed to provide life-long immunity. It has been hypothesized that the use of an effective vaccine in pigs would remove the source of tapeworm infection in humans, breaking the parasite’s life cycle and indirectly eliminating the causative agent of human neurocysticercosis [23].

The introduction of oxfendazole as a single dose therapy for porcine cysticercosis [25], coproantigen detection for improved diagnosis of taeniasis, and as already mentioned, a highly efficacious TSOL18 pig vaccine, have made control of transmission a potentially achievable goal. However, it remains unclear what would be the most effective method to apply these tools.

Oxfendazole has been shown to be highly effective against porcine cysticercosis, when given as a single dose at 30 mg/kg body weight. This is the minimal effective dose, that is, the dose that killed all parasites with no detectable side effects and left the meat fit for human consumption [26]. The inclusion of porcine treatment with oxfendazol in mass cysticercosis control programs is highly promising because it is a simple, effective, inexpensive, and potentially sustainable regime for decreasing the porcine reservoir of cysticercosis in disease-endemic countries.

The treatment of *T. solium*-infected pigs with oxfendazol as part of a control program for cysticercosis has the advantage of being relatively inexpensive, sustainable, and culturally acceptable [24]. Oxfendazole is superior to other agents for this purpose because it is nearly 100% effective and safe when given as a single dose. The main motivation was to find intervention strategies of vaccination and/or chemotherapy with which taeniasis/cysticercosis could be eliminated. By examining the literature on community interventions (for example references [17, 18, 22, 23]), we noticed that the ideal situation of having 100% effective treatments and vaccines as well as 100% of coverage is never attained. We then searched for successful strategies in non-ideal conditions which are more realistic in the field.

Minimal, sustainable intervention strategies to control *T. solium* infection have been proposed [27].

A mathematical model of the transmission dynamics of taenia-cysticercosis was formulated earlier [28]. The model comprises density-dependent equations for describing the flow of the parasite through the life cycle. From this model, we also derived a Susceptible-Infected (*SI*) model to describe the population of susceptibles and the prevalence of infection in humans and pigs. We showed that chemotherapeutic interventions against pig cysticercosis or against human taeniasis may reduce rapidly and effectively the mean intensity of human taeniasis, pig cysticercosis and human cysticercosis. However, once the treatment ceases, the infections return rapidly to their pre-control levels [28].

In this work, we now extend the *SI* model to incorporate susceptible individuals that can be vaccinated (pigs and/or humans). The model permits to vaccinate susceptible individuals and to treat infected population simultaneously, consecutively or in any order. The model permits to interrogate the dynamics of the infection under a plethora of interventions: for example, to vaccinate only pigs, to administer the vaccine twice, or to combine intervention strategies like pig vaccination and anthelmintic treatment to humans. The model can also examine different vaccination schedules under different regimes of repeated interventions, different protective vaccine efficacies, and different rates of coverage. The vaccine is applied to an initial population by varying coverage and efficacy rates of the vaccine, mimicking the coverage and effectiveness that can be covered in countries where the infection is intended to be controlled or eliminated.

We use the basic reproduction number *Ro* as derived in [28], and we examine the predictions of herd immunity. We have posed the following question: Would it be possible to find a vaccination schedule combined with chemotherapy to achieve elimination of the infection in pigs and humans? We present successful strategies for the elimination of the infection in pigs and humans from which a set of recommendations for the control and eventual elimination of the infection is given. Finally, we provide some guidelines to apply our model in realistic settings.

## Methods

### Mathematical model of vaccination against swine cysticercosis or human taeniasis

The mathematical model presented in [28] captures all essential aspects of the transmission dynamics of the infection of taenia-cysticercosis. This model is known in the literature of mathematical epidemiology as a density-dependent model that describes the flow of the parasite throughout the life cycle. However, this model does not allow us to deal explicitly with hosts individuals that are susceptible, immune or vaccinated. The model refers to worm loads of already infected individuals. Our interest now is to examine what could be the likely impact of the application of a vaccine administered to susceptible pigs or to susceptible humans upon the dynamics of the infection in pigs and humans. Derivation of the corresponding equations that allow for vaccination are presented in this section. This extended model is clearly designed to allow for the evaluation of different community interventions: application of a vaccine against either human taeniasis and/or swine cysticercosis and/or chemotherapeutic interventions against either the taenia and/or the larval cysticerci.

Developments of vaccines against helminth infections of humans has been the subject of much research. The impact of vaccination on the transmission dynamics of taenia/cysticercosis can be mirrored by the addition of an extra equation to represent an immune class within the human community.

Let us denote the size of the pig population as *N*_2_ = *J*_2_ + *S*_2_ + *V*_2_, where *J*_2_ is the number of infected pigs, *S*_2_ is the number of susceptible pigs and, *V*_2_ is the number of vaccinated pigs. Considering that the distribution of cysticerci among the pigs in a community can be described by a negative binomial distribution, the relationship between the proportion of infected individuals and the mean worm burden in a non-vaccinated population, is [28]:

$$ \frac{J_2}{N_2}=1-{\left(1+\frac{M_2(a)}{k_2}\right)}^{-{k}_2}=1-{\overset{\wedge }{S}}_2\left(\mathrm{a}\right), $$

where *k* represents inversely the degree of aggregation of the parasite, *M*_2_(*a*) is the mean intensity of cysticerci in pigs, and $\overset{\wedge }{S_2}(a)$ denotes the proportion of susceptible pigs at age *a*. Then the number of susceptibles at age *a* can be expressed as,

1 $$ {S}_2(a)={N}_2(a){\left(1+\frac{M_2(a)}{k_2}\right)}^{-{k}_2}. $$

If *N*_2_(*a*) = *N*_2_(0) exp(−*b*_2_*a*), where *N*_2_(0) is the initial population size of pigs, and *b*_2_ is the rate of mortality of the intermediate host (pigs), it follows that the rate of change of the number of susceptible individuals becomes [28]:

2 $$ \frac{dS_2(a)}{da}=-{S}_2(a)\left[{\left(1+\frac{M_2(a)}{k_2}\right)}^{-1}\frac{dM_2(a)}{da}+{b}_2\right]. $$

Now, if *c*_2_ denotes the vaccination coverage and *p*_2_ the protective efficacy of the vaccine, then *V*_2_(*a*) =  − |*c*_2_*p*_2_*S*_2_(*a*)| where *c*_2_, *p*_2_, and *S*_2_ are strictly ≥0, and || stands for the absolute value. This definition guarantees that the number of susceptibles does not increase along with the vaccinated individuals when the vaccine is applied. Thus, the equation that describes the vaccinated individuals is,

3 $$ \frac{dV_2(a)}{da}=-{V}_2(a)\left[{\left(1+\frac{M_2(a)}{k_2}\right)}^{-1}\frac{dM_2(a)}{da}+{b}_2\right]. $$

Hence, if we sum Eq. (2) and (3) we can get an equation that describes the effect of vaccination upon the mean intensity of pig cysticercosis, which is,

4 $$ \frac{dM_2(a)}{da}=-\left(1+{M}_2(a)/{k}_2\right)\left[{b}_2+\frac{1}{S_2(a)+{V}_2(a)}\left(\frac{dS_2(a)}{da}+\frac{dV_2(a)}{da}\right)\right] $$

The equations that describe the rate of change of susceptibles to human taeniasis (*S*_1_) and the rate of change of susceptibles to human cysticercosis (*S*_3_) are, respectively, [28]:

5 $$ \frac{dS_1(a)}{da}=-{S}_1(a)\left[{\left(1+\frac{M_1(a)}{k_1}\right)}^{-1}\frac{dM_1(a)}{da}+{b}_1\right] $$

6 $$ {\displaystyle \begin{array}{l}\frac{dS_3(a)}{da}=-{b}_1{N}_1(0)\exp \left(-{b}_1a\right)-{H}_3\left[\frac{dM_3(a)}{da}\right],\\ {}\ \end{array}} $$

where *N*_1_(0) is the initial human population size, and *b*_1_ is the rate of mortality of the definitive host (humans). The corresponding equations for describing the prevalence of human taeniasis (*J*_1_), the prevalence of pig cysticercosis (*J*_2_), and the prevalence of human cysticercosis (*J*_3_) are [28]:

7 $$ \frac{dJ_1(a)}{da}=-{b}_1{N}_1(0)\exp \left(-{b}_1a\right)-\frac{dS_1(a)}{da} $$

8 $$ \frac{dJ_2(a)}{da}=-{b}_2{N}_2(0)\exp \left(-{b}_2a\right)-\left(\frac{dS_2(a)}{da}+\frac{dV_2(a)}{da}\right) $$

9 $$ \frac{dJ_3(a)}{da}={H}_3\frac{dM_3(a)}{da}, $$

where *H*_3_ is the density of humans that can be infected with cysticerci, and *M*_3_(*a*) is the mean intensity of cysticerci in humans. For completeness, if we allow for vaccination against human taeniasis then Eq. (4) and Eq. (3) are replaced, respectively, by .

10 $$ \frac{dM_1(a)}{da}=-\left(1+{M}_1(a)/{k}_1\right)\left[{b}_1+\frac{1}{S_1(a)+{V}_1(a)}\left(\frac{dS_1(a)}{da}+\frac{dV_1(a)}{da}\right)\right] $$

11 $$ \frac{dV_1(a)}{da}=-{V}_1(a)\left[{\left(1+\frac{M_1(a)}{k{}_1}\right)}^{-1}\frac{dM_1(a)}{da}+{b}_1\right] $$

In summary, Eqs. (3) and (11) can be used to explore the effect of vaccination to susceptibles of pigs and humans, respectively; The impact of vaccination upon the mean worm intensity in pigs is given by Eq. (4), and the impact on the mean intensity of human taeniasis is given by Eq. (10). The prevalence of infection in humans taeniasis, pig cysticercosis, and human cysticercosis, are given by Eqs. (7), (8) and (9), respectively. Our model comprises 3 compartments: Susceptibles (*S*), Vaccinated (*V*), and Infecteds (*J* = *I*). Then we have an *SVI*−model.

### The basic reproductive number *Ro*

*Ro* is defined as the number of female offspring which are produced in average by one female parasite throughout her reproductive life span and which themselves survive to achieve sexual maturity in a population of *N* uninfected hosts, in other words, in the absence of density dependent constraints on worm survival. Thus *Ro* determines, in part, the level of parasitism in the population. In [28], we showed that *Ro* is equal to:

12 $$ Ro=\frac{\lambda {H}_1{H}_2{\beta}_1{\beta}_2{D}_1{D}_2}{\left({\mu}_3+{b}_2+{\alpha}_2+{\beta}_1{H}_1\right)\left({\mu}_2+{\beta}_2{H}_2+{\beta}_3{H}_3\right)\left({b}_1+{\mu}_1+{\alpha}_1\right)}. $$

where *λ* is the instantaneous per capita rate of egg production; *H*_1_ is the density of humans with *T. solium*; *H*_3_ is the density of humans with cysticerci; *H*_2_ is the density of pigs with cysticerci; *β*_1_ is rate of transmission of adult parasites; *β*_2_ is the rate of transmission of larvae to pigs; *D*_1_ is the proportion of parasites that reach sexual maturity; *D*_2_ is the proportion of parasites to reach infectivity in pigs; *μ*_1_ is the rate of mortality of the adult parasite; *μ*_2_ is the rate of egg mortality; *μ*_3_ is the rate of larvae mortality (pigs); *β*_1_ is the rate of transmission of adult parasites; *β*_2_ is the rate of transmission of larvae to pigs; *β*_3_ is the rate of transmission of larvae to humans; *α*_1_ is the severity of density-dependent constraints (taenia); *α*_2_ is the severity of density-dependent constraints (pigs). The range of feasible values of these parameters are given in [28]. We remark that the value of *Ro* for our model *SVI*−model is given by Eq. (12) since the *SVI*− model is directly derived from our density-dependent model [28]. Note that the density-dependent model elaborated in Part I [28], is embedded into the compartmental model without vaccination (*SI*− model), or with vaccination(*SVI*−model), and therefore the sensitivity analysis of the parameters is no longer required.

### Herd immunity

To eradicate a helminth infection by mass vaccination, the effective reproductive number *Ro* of the parasite must be less than unity in value. If, and only if, *Ro* is diminished to a value less than unity, then the immunization campaign can be rated as successful. To achieve this, the proportion *p*_*C*_ of the population that must be effectively immunized (assuming immune individuals are uninfected, and are therefore not able to produce transmission stages of the parasite) at any one time must satisfy the following condition [29–31]:

13 $$ {p}_C>1-\frac{1}{Ro} $$

Eq. (13) is also known as herd immunity, i.e., to eradicate a helminth infection, the critical proportion of the population that must be vaccinated is not necessarily 100%. In our simulations [28], *Ro* = 5.49 and Eq. (13) predicts that assuming a vaccine whose protective efficacy is 100% then the coverage rate must be *p*_*C*_ > 0.8178 ≈ 0.82. Eq. (13) also assumes that the vaccine provides life-long protection against infection by a helminth. Then a single program of mass vaccination or a short course of immunization would be necessary to protect the whole community. However, if the vaccine only gives protection for a few years, then a sizeable proportion of the population must be repeatedly immunized to sustain the necessary level of immunity for community protection. The reason for suggesting that any vaccine is unlikely to achieve life-long protection is associated with the observation that in natural helminth infections man appears, in general, to be unable to mount a fully protective immune response to reinvasion [32, 33]. If eradication is the aim of community immunization, the average age at vaccination must be less than that at which the infection is typically first acquired [29–31]. The relative merits of mass immunization compared to chemotherapy will depend critically on cost factors, since both forms of control may have to be administered repeatedly or sequentially (see below).

### Computer numerical simulation experiments of different vaccination strategies

The structure of the extended model permits the evaluation of the impact of different vaccination strategies upon the mean worm burden and, upon the populations of susceptibles, infecteds and vaccinated individuals (pigs and humans). Several complicated age-specific vaccination schedules can be handled. With other published models [34, 35] the equations and computations become rapidly messier. We can deal with a proportion of vaccinated individuals at birth or at a particular age or we can vaccinate a constant proportion at a constant rate. Here we present a very simple vaccination schedule: a constant proportion of susceptible individuals at age *a* are vaccinated.

Computer simulation experiments (CSE) were carried out to assess the impact of vaccination strategies on all the different compartments in which the population is divided.

The design of the CSE was as follows: no vaccination (control group) [28], or vaccination against pig cysticercosis only, or vaccination against human taeniasis only. In the latter strategy, it was assumed that the protective efficacy of the respective vaccine was 80% and the coverage rate was 80% (not shown). A fourth-order Runge-Kutta algorithm was used for solving the *SVI*−model. All the CSE were carried out by using the numerical differential solver ode45 in Matlab version R2008b, and in FORTRAN. Alternatively, the standard numerical differential equation NDSolve in Wolfram, Mathematica 2011, was also used. All computer programs rendered the same results and are available upon request. The computer programs are found in the Additional file 1 Matlab Programs. The programs are still not user-friendly, yet all the computer simulations carried out in this work can be reproduced. A global result that will become apparent is that vaccination against either pig cysticercosis or against human taeniasis will influence the transmission dynamics not only among vaccinees but also the dynamics of the other hosts as well.

## Results

### Intervention strategies to eliminate Taeniasis/Cysticercosis

In this section, we present different scenarios to eliminate taeniasis/cysticercosis. In all simulations, we used the same parameters as in [28].

#### First strategy: Application of two doses of vaccine against pig cysticercosis

**Fig. 1:**
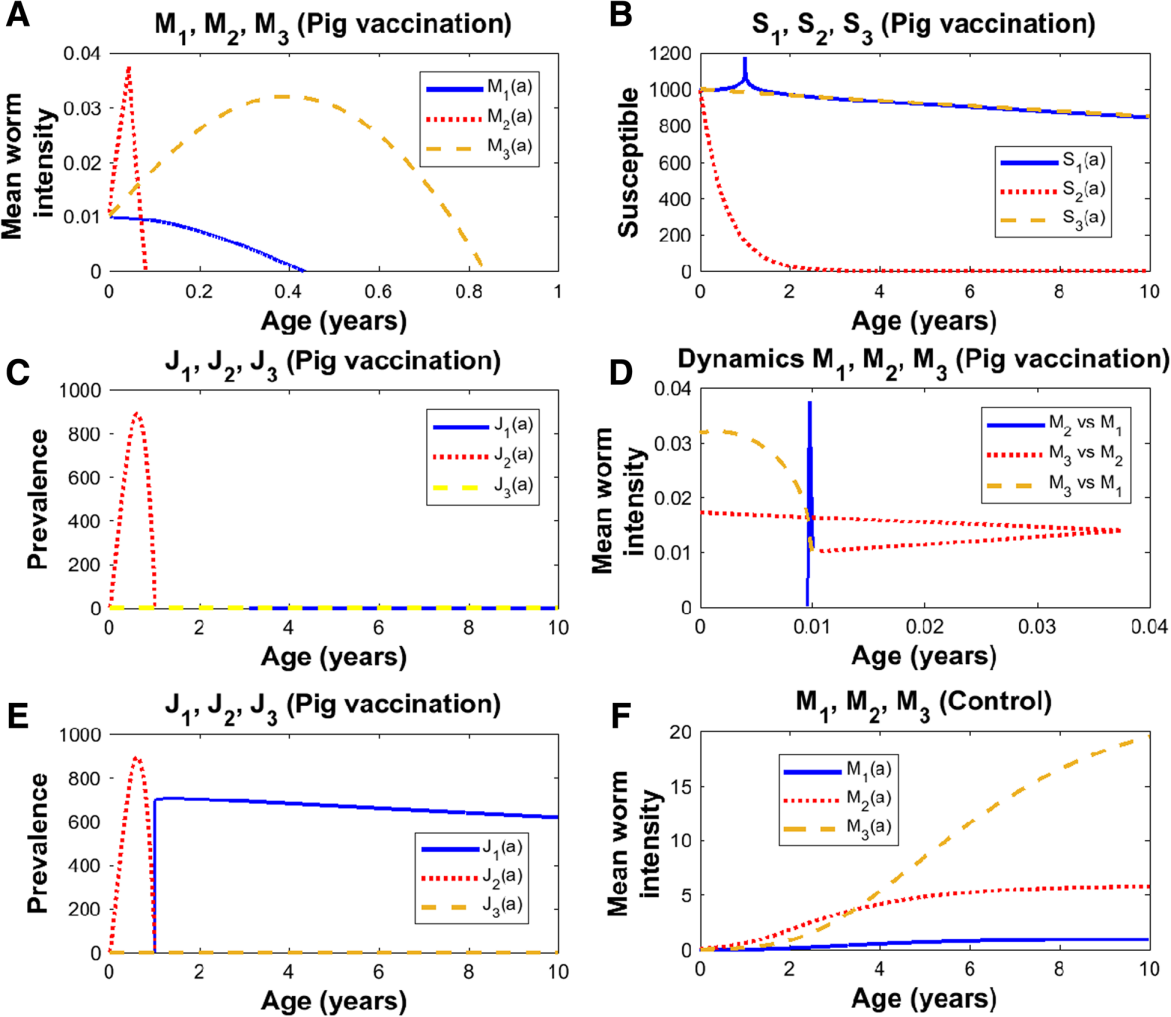
Two doses of vaccination with efficacy and coverage 100% (Table 1). In all graphs: human taeniasis (blue), human cisticercosis (yellow), human cisticercosis (red). In **a**, **b**, **c**, and **d** the vaccine is first applied to pigs of two weeks of age, and the second round is applied when pigs reach the age of 11 months with 25 days. In **e**, the 2 doses are applied but the second dose is administered to pigs of 11 months of age. In **f**, there is no vaccination. **a**) Mean worm intensities; **b**) Susceptibles; **c**) Prevalence of infected hosts; **d**) Phase spaces; **e**) Prevalence of infected hosts; **f**) Mean worm intensities in control. *Ro* = 5.49;  *p*_*C*_ = 0.82

Herein, two doses of the vaccine to pigs, assuming that both the protective efficacy of the vaccine and the coverage are 100% are administered (Table 1). The first round of vaccination is applied when pigs are one month of age and the second dose is applied to pigs of 11 moths plus 25 days (Table 1). Note that not only pig cysticercosis is eliminated but the infections in human taeniasis and human cysticercosis are also eliminated (Fig. 1a and c).

**Table 1:** Intervention strategies to eliminate taeniasis/cisticercosis. Vaccination against pig cystcercosis and human chemotherapy

Strategies	Vaccination to pigs					Chemotherapy				Efficacy (%)	Coverage (%)
						Pig		Human			
	Dose										
	1	2		3		1		1			
	Weeks	Month	Days	Month	Days	Month	Days	Month	Days		
Pig vaccination	2	11	25							100	100
Pig vaccination plus human chemotherapy and pig chemotherapy	1	12	9							100	82
						12	9			90	90
								12	9	90	90
Pig vaccination	0	1		12	24					82	82
Pig vaccination plus human chemotherapy	0	1		14	24					82	60
								14-16		90	90

If the second dose of pig vaccination is not applied, pig cysticercosis can be eliminated but there will be a rebound in the prevalence of human taeniasis and new cases of human cysticercosis will persist (Fig. 1e).

### Mean intensity

The control is presented in Fig. 1f, where the age-mean intensities of pig cysticercosis, human taeniasis, and human cysticercosis follow a hyperbolic pattern that each reaches an equilibrium (plateau). Following vaccination against pig cysticercosis (Fig. 1a), the mean intensity of taenia is reduced to zero in less than 5 months, the mean intensity of pig cysticercosis exhibits a dramatic decrease to zero as soon as the vaccine is applied and a triangle pattern emerges, and human cysticercosis display a concave pattern but it reaches the value of zero after 10 months. The rate of elimination of the mean intensities will depend upon the protective efficacy of the vaccine and the coverage rate.

### Susceptibles

The number of susceptibles to human taeniasis (*S*_1_), to pig cysticercosis (*S*_2_), and to human cysticercosis (*S*_3_), decrease as a function of age in the control group (Fig. 1b in José et al. [28]). Except for a transient spike of *S*_1_, pig vaccination scarcely influences both the values of *S*_1_ and *S*_3_ (Fig. 1b). When there is vaccination against cysticerci, *S*_2_ decreases exponentially and approaches to zero in less than 3 years. The latter behaviour is explained by the exponential decrease given by eq. (3). Recall that we are assuming coverage and vaccination rates of 80% and no chemotherapeutic intervention is made against the adult worm.

### Prevalence of infection

The prevalence of infection of human taeniasis (*J*_1_) in the control group increases with age until it reaches a plateau and eventually it starts to decline [28]. A similar pattern has been observed in natural communities [36]. The first time, pigs are vaccinated when they are 2 weeks old and the second time, they have 11 months plus 25 days (Table 1). The prevalence of pig cysticercosis (*J*_2_) shows an initial increase, reaches a maximum, and it rapidly declines to zero values around 1 year of age (Fig. 1c). If the second dose is delivered to pigs of age 11 months, there will be an abrupt increase in human taeniasis and new cases of human cysticercosis will continue to appear (Fig. 1e). At first glance, the increases of prevalence of human taeniasis (*J*_1_) and prevalence of pig cysticercosis (*J*_2_) following vaccination may seem a counterintuitive result. However, they occur because we are dealing with a Susceptible-Infected model in which a compartment of recovered individuals is not included. Vaccination reduces the number of susceptibles in the population so that they will move to the compartment of infected individuals with practically zero mean intensities. The prevalence of infection of human cysticercosis (*J*_3_) follows a hyperbolic pattern as has been observed in natural communities [37]. Because of vaccinating either pigs or humans *J*_3_ dies off near to zero even at very young ages (Fig. 1c).

### Phase spaces

In Fig. 1d, phase portraits of the dynamics of pig cysticercosis (*M*_2_) against human taeniasis (*M*_1_), human cysticercosis (*M*_3_) against pig cysticercosis (*M*_2_) and, human cysticercosis (*M*_3_) against human taeniasis (*M*_1_), are illustrated. Essentially, these phase spaces exhibit positive trajectories. When there is vaccination against pig cysticercosis (Fig. 1d) all phase spaces illustrate that *M*_1_, *M*_2_ and *M*_3_ are clearly diminished to zero. The phase space shows that the trajectories intersect at the point *M*_1_ = *M*_2_ = *M*_3_ = 0.01 which is given as an initial condition. We remark that this point acts as an attractor from the mathematics of the dynamics of the system, and it defines the minimum level of infection attained by the effects of the vaccination strategies that tend to extinguish the infections in pigs and humans. In practice, this should be considered a successful vaccination strategy.

When there is vaccination against pig cysticercosis, the trajectory of *M*_2_ versus *M*_1_ follows a vertical decline to zero with a fixed value of *M*_1_ (Fig. 1d). Note that there are negative relationships between *M*_1_ or *M*_2_ with *M*_3_ (Fig. 1d). A slight decrease of *M*_1_ or *M*_2_ is related to a fast decrease in *M*_3_ (Fig. 1d). The vaccination strategy leads to zero mean intensities and the phase spaces highlights the routes to extinction.

#### Second strategy: Application of two doses of vaccine against pig cysticercosis with coverage less than 100%

**Fig. 2:**
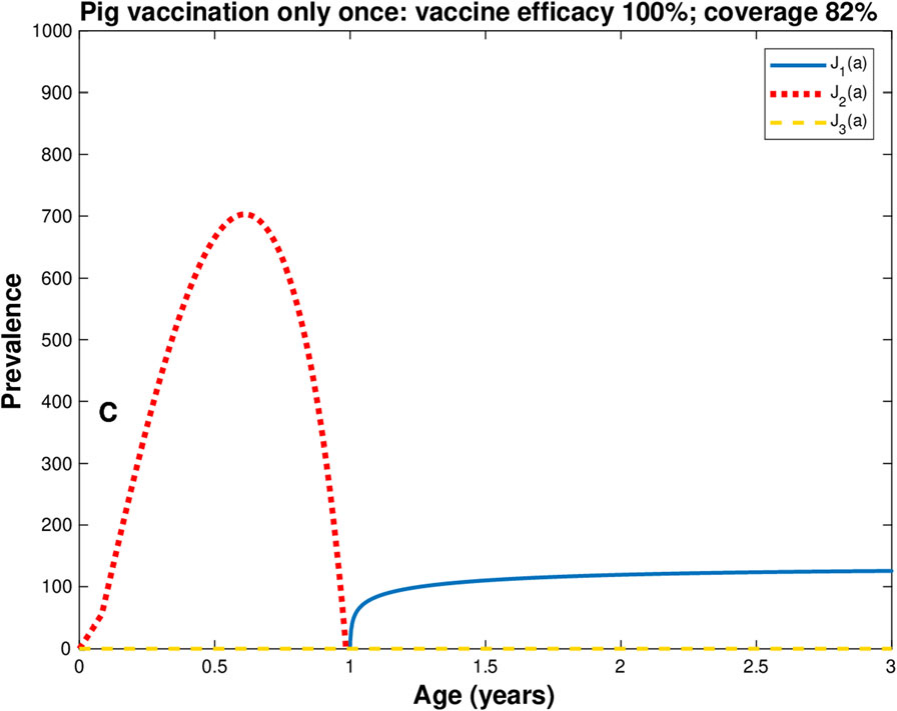
Prevalence of infection in pig (line red), human cysticercosis (line yellow), and human taeniasis (line blue) with vaccine efficacy equal to 100% and coverage at 82%. Only one dose of vaccination to pigs of two weeks old. *Ro* = 5.49;  *p*_*C*_ = 0.82

If we now assume a vaccine efficacy of 100% but the coverage is reduced to 82%, the result of pig vaccination is that pig cysticercosis is eliminated as predicted by Eq. (13), but there is an unexpected increase in the prevalence of human taeniasis (Fig. 2). In the next sections we present different interventions with which this goal can be attained.

#### Third strategy: Application of two doses plus chemotherapy against pig cysticercosis and human taeniasis

**Fig. 3:**
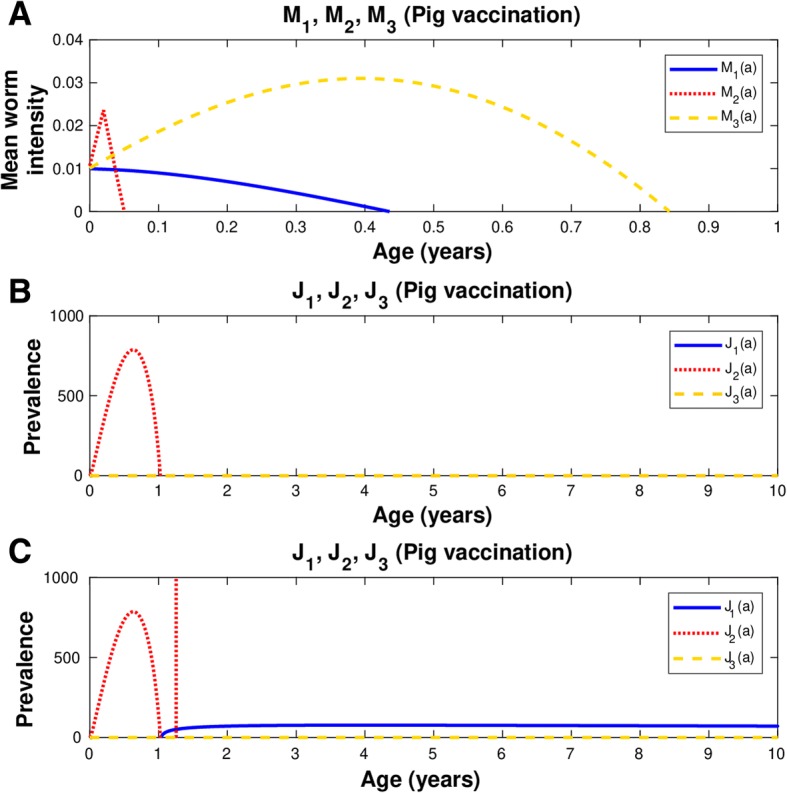
Vaccine efficacy is 100% and coverage rate is 82%. In all graphs: human taeniasis (blue), human cisticercosis (yellow), human cisticercosis (red). In **a** and **b**, the vaccine is applied to pigs of one week of age and the second dose is given later to pigs of 12 months and 9 days of age. Simultaneously, chemotherapy is applied to both pigs and humans. In **c**, the same immunization protocol is followed as in **a** and **b**, but no chemotherapy is applied. **a**) Mean worm intensities; **b**) Prevalence of infected hosts; **c**) Prevalence of infected hosts. The coverage rate and the efficacy of the anthelmintic drugs in pigs and humans is 90 and 90%, respectively. *Ro* = 5.49;  *p*_*C*_ = 0.82

The mathematical model allows to predict what happens with the infection in pigs and humans under different intervention schemes, and it is also possible to know when we need to apply the second or any course of vaccination. In this case, a second dose of vaccination is needed at the exact moment when the prevalence of pig infection arrives at zero, Otherwise, the infection in human taeniasis will not disappear (Fig. 3c).

##### Case 1

If the vaccine efficacy is 100% but the coverage is reduced to 82%, the vaccination schedule must change. In this case we applied the vaccine against pig cysticercosis twice. The first dose is administered when pigs have one week of age and the second dose is applied to pigs of 12 months and 9 days old (Table 1). The strategy is effective only if chemotherapy is applied simultaneously and constantly to both pig and humans with respective drug efficacies of 90% (Table 1). This combined strategy of vaccination and chemotherapy is effective since human taeniasis, and human and pig cysticercosis are eliminated (Fig. 3a and b).

##### Case 2

**Fig. 4:**
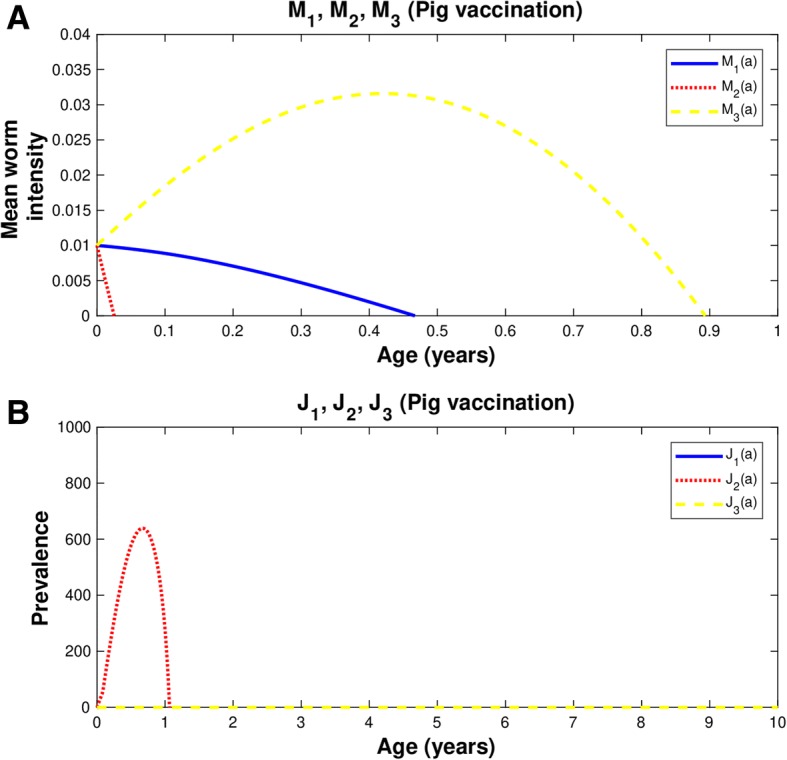
Vaccine efficacy 82% and coverage 82%. The vaccination strategy includes three doses: the first dose is given to newborn pigs, the second is applied to pigs of one month of age, and the third dose is for pigs with 12 months plus 24 days of age. In all graphs: human taeniasis (blue), human cisticercosis (yellow), human cisticercosis (red). **a**) Mean worm intensities; **b**) Prevalence of infected hosts. *Ro* = 5.49;  *p*_*C*_ = 0.82

If both the efficacy and coverage are equal to 82%, the first dose is applied to pigs at birth, the second dose is administered when pigs are one month of age, and the third dose is applied to pigs of one year and 25 days old (Fig. 4).

##### Case 3

**Fig. 5:**
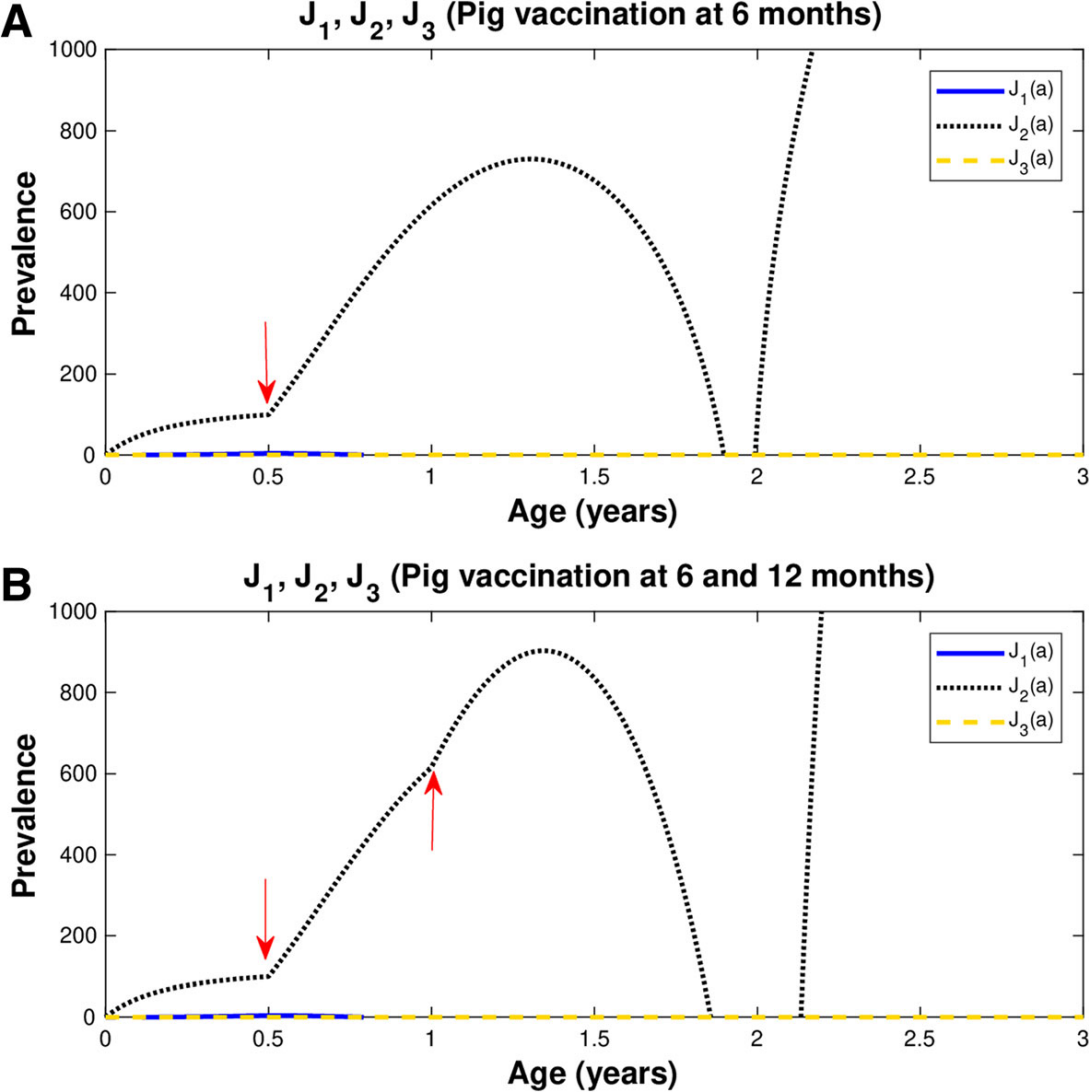
Vaccine efficacy is 100% and coverage rate is 82%. Prevalence of infection in pigs (black-dotted line). Vaccination is applied to: **a**) pigs at 6 months of age (red arrow) **b**) pigs at 6 and 12 months of age (red arrows). *Ro* = 5.49;  *p*_*C*_ = 0.82

Considering the short life span of pigs, we simulated the case in which we apply one round of vaccination to pigs at 6 months of age (red arrow) (Fig. 5a), and two rounds at 6 and 12 months of age (red arrows) (Fig. 5b). We are assuming a protective efficacy of 100% and a coverage rate of 82% in both cases. Note that in both cases, the prevalence of pig cysticercosis increases after the application of the vaccine at 6 months of age. When a second vaccination is administered to pigs of one year of age there is a further increase in pig prevalence. In both strategies, the prevalence reaches a maximum despite the vaccinations, and the prevalence starts to decline to values near to zero. The decrease is faster when two vaccinations are applied than when only one vaccination is given. In the latter case (A), this occurs at age less than two years of age, and in the former case it happens even at an earlier age (B). However, there is a dramatic increase in the prevalence of pig cysticercosis at 2 years of age in case (A) and at age slightly higher than 2 years of age in case (B). The age interval between the prevalence near to zero and the dramatic increases in the prevalence of pig cysticercosis is larger in case (B) than in case (A). Then, both vaccination regimes lead to a sudden rebound in the prevalence of pig cisticercosis.

**Remark**: In Table 1 of the manuscript Part 1 [28], we set the rate of mortality of the intermediate host *b*_2_ in the range 0.8 − 1.15, which means that the life span of pigs lies between *b*_2_^−1^ ∈ [0.87 − 1.15] years of age. There are no pigs older than *b*_2_^−1^. If it seems so, is due to a constant rate of replenishment of susceptible pigs to a population of constant size *N*_2_. Then, for example, pigs of “2 years of age” were actually born 2 − *b*_2_^−1^ years ago.

#### Fourth strategy: Three vaccination doses against pig cysticercosis and one round of chemotherapy to human taeniasis

**Fig. 6:**
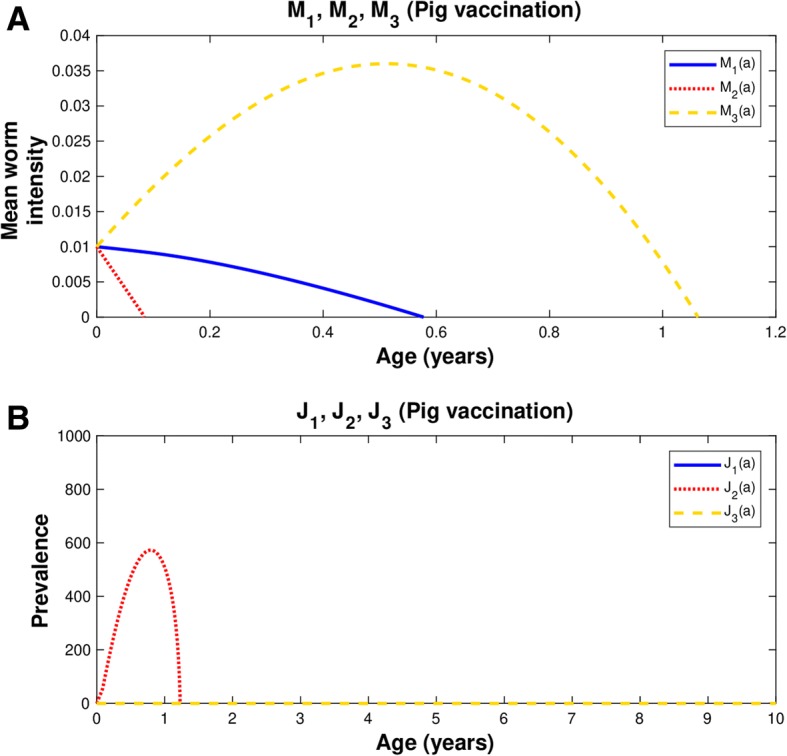
Vaccine efficacy 82% and coverage 60%. In all graphs: human taeniasis (blue), human cisticercosis (yellow), human cisticercosis (red). The vaccination strategy includes three doses: the first dose is given to newborn pigs, the second is applied to pigs of one month of age, and the third dose is for pigs with 14 months plus 24 days of age. Concomitantly to the vaccination strategy for pigs, chemotherapy against human taeniasis is administered to humans of 14 and 16 months of age. **a**) Mean worm intensities; **b**) Prevalence of infected hosts. *Ro* = 5.49;  *p*_*C*_ = 0.60

If we assume a vaccine efficacy of 82% and a coverage rate of 60%, the intervention strategy is as follows: First, we employ the vaccination when pigs are zero-month-old and when pigs are one month old, and we use the third dose when pigs are one year and 24 days of age (Table 1). We determined the age of the second dose by examining the age at which the prevalence of the pig infection is close to zero. A chemotherapeutic treatment is also applied to human with taeniasis, when pigs received the third dose of vaccination (Fig. 6 and Table 1). The treatment is administered to 90% of infected individuals, with a drug efficacy of 90%. This intervention schedule must be used because if chemotherapy is not applied, the infection will be greater in pigs with cysticercosis and human with taeniasis (not shown).

Overall the mean worm intensity for human taeniasis and pig and human cisticercosis attain the value of zero when the efficacy of the vaccine is 100% (Figs. 1, 2, 3 and 4). Quantitatively the rate of decline of the mean worm burdens depends on the percentage of vaccine efficacy and the coverage rate. The closer the vaccine efficacy is to 100% the faster is the decline. Note that human susceptibles to taeniasis (*S*_1_) display an abrupt peak at about 1 year of age; susceptibles to pig cisticercosis may decline exponentially (Fig. 1) or faster than exponentially (Figs. 2, 3 and 4). The behaviour for the prevalence of infection (*J*) is practically the same for all cases: there is an increase of *J*_2_ up to 6.7 months and a rapid decline thereafter reaching the value of zero at about a year or slightly more than one year of age. For all these strategies eradication is achieved.

Table 1 summarizes the details of the four strategies and their results. The combined use of pig vaccination and chemotherapy against human taeniasis stands out as one of the more effective interventions than any other strategy. The combined strategies become even more important when the coverage rates are of the order of 60–80%, and vaccine and drug efficacies are as low as 60 and 90%, respectively.

## Discussion

In this work, we have shown that there is a panoply of different mass intervention strategies with which it is possible to eliminate taenia/cysticercosis. We emphasize that our model is general in its application. Our model allowed us to assess the impact of combined human-pig intervention upon the infection of *T. solium* in humans and human cysticercosis. The application of the model is not restricted to a community or a country. It can be applied worldwide but not with a single strategy. To test the validity of the model, we first showed that by vaccinating the 100% of the pig population with a 100% effective vaccine it is possible to eliminate pig and human cysticercosis and human taeniasis. In practice, vaccination against pig cysticercosis and/or chemotherapy interventions against human taeniasis, pose several caveats. First, the number of individuals with taeniasis is not only small but they are very difficult to encounter and to diagnose. The rate of compliance will be affected by several factors including culture [22]. We examined different strategies in which either the efficacy of the vaccine against pig cysticercosis and/or the coverage of drug treatment are less than 100%. If the vaccine efficacy is 100% but the coverage of pig vaccination is 82%, pig cysticercosis will be eliminated as predicted by herd immunity. Yet there will be an increase in human taeniasis. Albeit counterintuitive, there could be increases in both human taeniasis prevalence (*J*_1_) and pig cysticercosis prevalence (*J*_2_) following vaccination (Fig. 2). The strength of our model comes from the fact that we derived two compartmental models, to wit, a Susceptible-Infected model (*SI*) [28] and a Susceptible-Vaccinated-Infected model (*SVI*), which have embedded a density-dependent model of the actual life cycle of the parasite [28]. Consequently, the basic reproductive number *Ro* (Eq. 12) is one and the same for the density-dependent model [28] and for both compartmental models. With the *SVI*−model we succeeded to find different effective intervention strategies to interrupt the transmission dynamics in both humans and pigs. If the vaccine efficacy is of the order of 82% and the coverage rate is 82%, our model predicts that the successful strategy is to apply 3 doses of vaccine to piglets of zero-month, one month of age plus a third dose administered at 1.065 year of age (Fig. 6). In the worst-case scenario, with a vaccine efficacy of 82% and a coverage rate of 60%, the successful strategy consists of three doses of pig vaccination, one applied to newborn pigs, the second dose to pigs of one-month of age, and the third dose is given to pigs of 1.22 years of age, *plus* chemotherapy against human taeniasis for the human population that reach the age between 14 to 16 months of age (Fig. 6). We remark that the second dose is in general applied in the age window in which the prevalence of infection among the pig population is approaching to zero. According to our results, we would recommend applying the second dose of the vaccine in the critical age window and before the slaughtering of pigs. Otherwise, there could be a dramatic increase of human taeniasis and perhaps of human cysticercosis (Fig. 5). Our proposal implies to postpone the slaughtering of sows 2–3 months after the second round of immunization. The effects of some strategies, not only are counterintuitive but also counterproductive. These examples illustrate the importance of our model in designing effective vaccination strategies. Any program of intervention must contemplate the need of estimating several parameters of the target population. In all cases it is mandatory to perform diagnostic test for human taeniasis and pig cysticercosis. To apply our model, it is necessary to estimate the population densities of both human and pig populations, the efficacy of both the vaccine and the anthelmintic drug, and the coverages of vaccination and drug treatment. Once the parameter Ro is estimated, it is necessary to define the level of coverage, the vaccination efficacy, and the age span of eligible pigs for vaccination. If two or more doses of pig vaccination will be applied, it is crucial to determine the age of the pig at which the second dose must be administered. Everyone interested in eliminating the infection locally or globally, can use our model to simulate those strategies of only pig vaccination or pig vaccination combined with pig and/or human chemotherapy. The predictions of our mathematical model with vaccine agree with those reported in the literature [17, 18, 22]. Most of the interventions that have been tested for *T. solium* to date have relied on the treatment of the definitive hosts (humans) to remove tapeworm infections [24] and this may have increased the overall susceptibility of the pig population to cysticercosis. Indeed, one study found an increase in the prevalence of porcine cysticercosis after mass treatment of the human population with a taeniacide [38]. Ideally, the best way to fight the infection is to vaccinate 100% of the pigs with a vaccine with 100% efficacy. However, in practice, it will not be possible to achieve 100% of coverage in all communities and the vaccine may not be 100% efficacious. Then the combination of vaccine and chemotherapy turns out to be the best option to eliminate the infection even with coverage 60% and efficacy of 82%. If it is not possible, coverage and efficacy of 60% could be applied, but for controlling purposes. Implementing campaigns to inform the population in combination with the vaccination of pigs and chemotherapy to humans is the best strategy to combat taeniasis/cysticercosis in any country, even though it generates high costs in a short time, the health problems caused by the infection will be reduced and it will be eliminated. An advantage of using a combination of pig vaccination plus chemotherapy against human taeniasis, in comparison to using vaccination alone or chemotherapy alone, is that complete elimination of the infection in both pigs and humans can be attained. To achieve WHO goals, we think that different interventions should be sustained on a wide scale.

It has been suggested that if a vaccine for cestodes were available and if the immunity changed rapidly, then the endemic steady state may be destabilized, resulting in oscillations in the parasite population [39]. In our CSE, the inspection of the phase spaces does not seem to support that notion. Previous models of *T. solium* transmission rely on assumptions about the biology of *T. solium* that must be made due to the absence of definitive scientific data in many areas, for example, the survival time for eggs in the environment [34, 35]. Future improvements in the vaccine, in relation to minimizing the number of exposures to the recombinant antigen required to induce/maintain protection and changing the method of delivery from parenteral to an oral route, would enhance the ease with which the vaccine could be applied in undertaking *T. solium* control.

It could be anticipated that a combination of both vaccination/oxfendazole treatment of pigs together with anthelmintic treatment of the human population to eliminate the adult tapeworms, particularly when control procedures were first implemented, would have the greatest and most rapid impact on reducing the incidence of neurocysticercosis [27, 39, 40].

Our model has the potential for designing optimal strategies to achieve the control, reduction, or to eliminate the infection. In practice, the relative efficacy of interventions needs to be demonstrated by their implementation and careful evaluation in field settings and by undertaking cost/benefit analyses based on the outcomes. It is not realistic to imagine that *T. solium* control measures would be implemented indefinitely. With our model rational predictions can be made about how rapidly *T. solium* transmission would be re-established in a region where transmission through both humans and pigs had been interrupted. Re-establishment of the parasite would be affected by the prevalence of *T. solium* transmission in surrounding regions, the size of the area where control had been achieved, and the rates of migration of humans and pigs from endemic areas.

The stochastic version of our model, without assuming an overdispersed distribution of the parasites, is an ongoing work in our group. We are also developing an automatic computational workflow to design intervention strategies aimed to the control and/or eliminate the burden of the infection.

Here we have presented effective interventions aimed to eliminate the transmission dynamics of pig and human cysticercosis and human taeniasis. We have shown how to elaborate intervention strategies that have the potential to reduce or eliminate human neurocysticercosis, pig cysticercosis, and human taeniasis. The latter are the goals established by the WHO [10]. We hope that our present work may contribute to that target.

## Conclusions

A mathematical model of taenia-cysticercosis that divides the population into susceptible, infected, and vaccinated individuals is formulated. The model is based upon the life cycle of the parasite. Our mathematical model has the potential for the effective design and implementation of mass vaccination and/or chemotherapeutic treatment to eliminate pig cysticercosis, human taeniasis and human neurocysticercosis. The model can be adapted to any given community with mild, moderate endemicity, or even in hyperendemic regions.

## Additional file

Additional file 1: Matlab Programs. (RAR 6 kb)
